# Developing a Complex Intervention Plan for Physical Activity in Overweight and Obese Endometrial Cancer Patients: A Multimethod Study

**DOI:** 10.1155/jonm/3951371

**Published:** 2026-03-04

**Authors:** Yongli Wang, Jingying Yang, Jianliu Wang, Jingjing Gong, Yuanyuan Gong, Qingran Li, Dandan Yang, Hongmei Zhu, Peng Liu, Yiqian Chen, Yingchao xiong, Xiaodan Li

**Affiliations:** ^1^ Department of Paediatrics, The People’s Hospital of Peking University, Beijing, China; ^2^ Nursing School of Peking University, Beijing, China; ^3^ Department of Obstetrics and Gynaecology, The People’s Hospital of Peking University, Beijing, China, unicamp.br; ^4^ Department of Nutrition, Peking University People’s Hospital, Beijing, China, pku.edu.cn; ^5^ Department of Endocrinology, Peking University People’s Hospital, Beijing, China, pku.edu.cn

**Keywords:** complex intervention, endometrial cancer, MRC framework, overweight and obese, physical activity

## Abstract

**Aims and Objectives:**

This study aims to develop a comprehensive physical activity (PA) intervention tailored for overweight and obese patients with endometrial cancer (EC). It integrates theoretical frameworks, empirical evidence, expert opinions, and stakeholder perspectives to assist clinical providers in implementing standardized PA guidelines.

**Background:**

Obesity is a significant risk factor for EC and is closely linked to treatment outcomes. Although previous studies have focused on the role of PA in weight loss and survival, they lack detailed, evidence‐based guidance specifically tailored for EC patients.

**Design and Methods:**

We developed a PA instruction program using the Evidence‐Based Nursing Practice Pathway and the Medical Research Council’s Framework for Complex Interventions. The development process consisted of three phases: a preparatory phase, the development and implementation of evidence‐based interventions, and the refinement of the program through Delphi consultation.

**Results:**

The program framework was developed using multiple methods, including evidence searches (guidelines and expert consensus), semistructured interviews, and expert consultations. It encompasses preinstruction, implementation methods, and strategies for maintaining PA. Instructional materials such as posters, brochures, videos, and checklists were created to facilitate clinical integration.

**Conclusions:**

This study is the first to develop a tailored PA program for overweight EC patients. The rigorous design, based on complex intervention frameworks and expert input, has the potential to improve clinical practice, enhance tumor remission rates, and improve fertility outcomes.

**Relevance to Clinical Practice:**

Utilizing the MRC framework, this study integrated evidence and stakeholder feedback to develop a tailored intervention program. It aims to increase PA among overweight and obese EC patients by providing educational materials for clinicians and patients, thereby promoting its adoption in clinical practice.

**Trial Registration:** ClinicalTrials.gov_identifier: NCT06312917

## 1. Background

Endometrial cancer (EC) is recognized as the sixth most prevalent malignant tumor among women globally. In 2022, it was reported that over 427,000 women were newly diagnosed with this malignancy [1], highlighting its significant threat to women’s health due to its alarming epidemiological trend. In cases of early‐stage EC where fertility preservation is desired, clinical guidelines recommend oral progestogen–based therapy as the preferred conservative treatment over total hysterectomy [2]. The primary goal of this approach is to manage the disease while preserving the reproductive capabilities of the patients [3, 4]. A key measure of this therapy’s success is the complete response, characterized by the total regression of endometrial lesions and stromal decidualization, as confirmed through posttreatment histopathological examination.

However, oral progestogen therapy often leads to adverse effects, such as increased appetite, which can result in patients becoming overweight or obese during treatment [5]. This weight gain exacerbates the risks associated with both the efficacy of the treatment and the long‐term prognosis of the patients [6, 7]. Substantial research has shown that EC patients with overweight or obesity undergoing fertility‐sparing therapy not only face a higher risk of disease recurrence but also encounter significant challenges in achieving complete remission following progestogen administration [8, 9]. A systematic review has further indicated that a higher body mass index (BMI) in EC patients is associated with longer surgical durations and an increased incidence of postoperative complications [10]. Posttreatment, obese survivors of EC continue to face a compromised prognosis, reduced quality of life, and elevated mortality rates [11, 12]. These findings collectively emphasize the importance of implementing effective weight management strategies to optimize treatment outcomes and improve the long‐term prognosis for EC patients.

Physical activity (PA) is fundamental to weight management and has been extensively documented for its benefits in cancer patients [13, 14]. Mechanistically, exercise aids in weight control by enhancing energy expenditure and improving musculoskeletal health, reducing blood pressure and cholesterol levels, and increasing insulin sensitivity [15]. Additionally, PA can diminish the release of inflammatory mediators and tumor markers, relieve cancer‐related fatigue, and offer multidimensional health benefits to cancer patients [16]. Notably, existing studies have confirmed that moderate PA not only boosts pregnancy rates in EC patients undergoing fertility‐sparing treatment [17] but also positively affects disease progression and prognostic outcomes [18].

Despite the recommendation from the American College of Sports Medicine (ACSM) that cancer survivors should engage in at least 150 min of moderate‐to‐vigorous aerobic and resistance exercise per week, with increased duration and intensity suggested for obese patients [19], clinical evidence indicates that only a small proportion of overweight or obese EC patients meet these guidelines [5]. The lack of adherence can be attributed to several factors, including the absence of exercise programs tailored to specific types and intensities, insufficient consideration of patient preferences, low participation rates, and the inconsistent quality of related studies. Critically, there is a global lack of standardized exercise guidelines tailored specifically for EC patients, which significantly impedes the effective translation of evidence‐based PA recommendations into clinical practice.

The Medical Research Council (MRC) developed a framework for evaluating complex interventions, which was updated in 2021 [20]. This revised framework incorporates additional core elements that guide the development and evaluation of complex interventions. It covers stages such as development, feasibility assessment, intervention design, and formal evaluation. Research indicates that interventions guided by the MRC framework are extensively utilized in healthcare and social services. They also provide standardized approaches for addressing clinical nursing issues and enhancing the quality of nursing care [21, 22].

Consequently, this study adheres to the recommendations for developing complex interventions outlined in the MRC framework. It examines the significant role of PA in this context and develops an intervention program specifically for overweight and obese EC patients. The objective is to improve the efficiency of weight management and provide theoretical support for enhancing patient outcomes.

## 2. Methods

**FIGURE 1:**
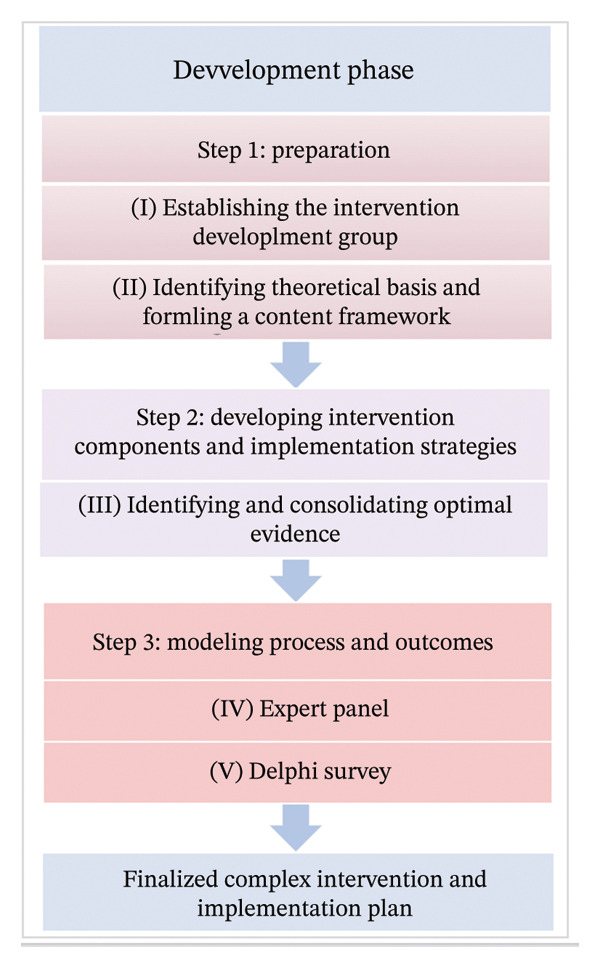
Flowchart for the development process of a complex physical activity intervention plan for overweight and obese patients with endometrial cancer.

A multimethod study was carried out based on the development phase elements of the MRC complex intervention framework. The study encompassed three steps:

 Step 1: Preparation Phase
 An intervention development team was organized. Moreover, an evidence‐based nursing practice pathway model was selected. Then, qualitative interviews were conducted with relevant stakeholders.
 Step 2: Intervention Design and Implementation Strategies
 The best evidence for PA was integrated into the intervention design.
 Step 3: Modeling Process and Outcomes
 Through a series of group discussions, a PA guidance program specifically tailored for overweight and obese EC patients was drafted and refined. Figure 1 illustrates the development process of the Complex PA Intervention Plan for Overweight and Obese Patients with EC.

### 2.1. Methods—Step 1: Preparation

#### 2.1.1. Establishment of an Intervention Development Team

Developing a rigorous and comprehensive intervention plan necessitates a structured intervention team. Following team‐building principles, the PA intervention program for overweight and obese EC patients was developed by an interdisciplinary team. This team consisted of experts in obstetrics and gynecology, kinesiology, evidence‐based medicine, management, and other relevant stakeholders. The team was engaged throughout the development process, including the implementation phase of the final intervention.

#### 2.1.2. Selection of the Theoretical Basis and Formation of the Content Framework for the PA Program

Identifying relevant theoretical frameworks is crucial in the development of complex interventions. During this phase, literature reviews and stakeholder interviews were conducted to select an appropriate theoretical model that underpinned the content framework of the PA guidance program. The “Pathway for Evidence‐Based Nursing Practice” was chosen as the theoretical framework based on the literature reviewed [23]. This research synthesized the best evidence for weight management in overweight and obese EC patients and utilized the Delphi method to develop a PA program. This approach provides a scientifically robust reference for the translation into future clinical practice and offers a theoretical basis for implementing the program. Following the existing research, the theoretical program will be introduced into clinical settings for evidence application, with an aim of developing a standardized PA management process to guide clinical practice for overweight and obese EC patients.

#### 2.1.3. Descriptive Study of Stakeholders

The qualitative descriptive study aimed to explore the background factors related to PA experiences among overweight and obese EC patients. Specifically, the study focused on identifying the facilitators and barriers encountered by EC patients during PA, as well as the management‐related needs for PA practices.

#### 2.1.4. Study Design

A semistructured descriptive qualitative study was conducted with overweight and obese EC patients by an intervention team.

#### 2.1.5. Study Population, Recruitment, and Sampling

A purposive sampling method was used to select participants from a tertiary hospital in Beijing. These participants were overweight or obese EC patients currently undergoing weight management from November 2022 to May 2023. The inclusion criteria included being diagnosed with EC, currently engaged in a PA intervention, possessing good language comprehension and communication skills, being overweight or obese, and having voluntarily participated and signed an informed consent form. Exclusion criteria included severe mental illness preventing participation, inability to cooperate with the study, and failure to participate throughout the study period for personal reasons. A maximum variation sampling technique, a submethod of purposive sampling, was employed to select the final sample of 17 EC patients.

#### 2.1.6. Data Collection and Processing

The Consolidated Criteria for Reporting Qualitative Research (COREQ) checklist [24] was used as a guide for reporting this study. The interview protocol was developed based on a literature review, expert consultations (involving one gynecologist, one rehabilitation specialist, and three oncology nursing experts), and a preliminary survey. The final version of the interview protocol is presented in the supplementary material. We carried out interviews in a quiet hospital meeting room. All sessions, including observations of expressions and body language, were recorded on‐site. All interviews were conducted in Chinese and analyzed through NVivo 14.23.0 transcription software. Following the Declaration of Helsinki, all data collection was anonymous, used solely for academic research, and ensured patient privacy through data protection measures.

#### 2.1.7. Data Analysis

This study used the seven‐step data analysis procedure proposed by Colaizzi [25]. An inductive technique was utilized to gradually refine and summarize the data, starting with the most fundamental information. The interview data underwent thematic analysis with an emphasis on the real‐life experiences of overweight and obese EC patients regarding PA. An experienced researcher (YW), certified in qualitative research, developed the initial coding draft. The second researcher (JY) reviewed and tested the codes. Discussions were undertaken to enhance their reliability. In the event of discrepancies between YW and JY, a third experienced researcher was consulted to resolve the issues, resulting in the final coding themes.

### 2.2. Methods—Step 2: Developing Intervention Components and Implementation Strategies

#### 2.2.1. Summary of the Best Evidence

##### 2.2.1.1. Evidence Retrieval and Synthesis

We conducted a search and synthesis of evidence, focusing on the content framework to integrate high‐quality and readily available evidence. This process involved a systematic search, screening, and evaluation of the literature pertaining to PA in overweight and obese EC patients. The PIPOST model, developed by the Evidence‐Based Nursing Center at Fudan University, guided the formulation of the evidence‐based research question. The population (P) included overweight and obese EC patients, defined using the following criteria: (1) For Chinese literature, the Chinese obesity classification standards were applied: overweight as a BMI of 24.0–27.9 kg/m^2^ and obesity as BMI ≥ 28.0 kg/m^2^, reflecting the body composition characteristics of the Chinese population. (2) For English literature, the World Health Organization (WHO) international standards were used: overweight as BMI 25.0–29.9 kg/m^2^ and obesity as BMI ≥ 30.0 kg/m^2^. (3) All included patients were diagnosed with EC through postoperative histopathological examination, following the 2021 International Federation of Gynecology and Obstetrics (FIGO) Staging Criteria for Endometrial Carcinoma. The exclusion criteria included the following: (1) patients with other systemic malignancies or a history of malignant tumors; (2) patients with severe dysfunction of vital organs (heart, liver, and kidney) who could not tolerate treatment or complete follow‐up; (3) patients who received neoadjuvant radiotherapy or chemotherapy before surgery; and (4) patients with missing key clinical data or lost to follow‐up during the study. The intervention (I) was PA. The professionals (P) involved included gynecologic oncologists, specialized nurses in gynecologic oncology, oncology rehabilitation therapists, and registered clinical dietitians. The outcomes (O) measured were weight, BMI, and other related metrics. The settings (S) were either hospitals or homes. The types of evidence (T) included published clinical guidelines, systematic reviews, evidence summaries, expert consensus, and original research in both Chinese and English. This evidence summary was registered with the “National Evidence Summary Registration Platform” of the Fudan Evidence‐Based Nursing Center prior to the study and received approval. The registration number was ES20232492.

##### 2.2.1.2. Data Sources and Search Strategy

Relevant guidelines, professional association websites, and domestic and international literature databases, such as Cochrane, JBI Evidence‐Based Healthcare Database, ClinicalKey for Nursing, Medline, PubMed, Embase, CINAHL, CNKI, CBM, and Wanfan, were utilized for the evidence search. We used a top–down approach based on the “6S” evidence resource model to search for literature related to PA in overweight and obese EC populations. The search terms encompassed both Chinese and English keywords, including “endometrial cancer,” “gynecological cancer,” “cancer,” “tumor,” “overweight,” “obesity,” “physical activity,” “exercise,” “exercise therapy,” “cancer survivor,” “neoplasm,” “endometrial neoplasms,” and “endometrial atypical hyperplasia.” The search was conducted in relevant guideline websites, professional association sites, and databases from the inception of each database until June 2023. The supplementary material presents detailed search strategies and resources.

##### 2.2.1.3. Literature Screening, Evidence Extraction, and Evaluation

Upon completion of the literature search, all results were imported into EndNote 20.0 to check for duplicates. Two researchers (YW and JY), both trained and certified in JBI systematic review methodology, aimed to include all publicly accessible research on PA interventions for overweight or obese EC patients, restricted to publications in Chinese or English. The exclusion criteria were as follows: (1) outdated guidelines that have since been updated, (2) publications that do not align with the research focus, (3) interpretations or commentary on guidelines, (4) Chinese translations of international guidelines or original articles, (5) publications that were not accessible in full through any available channel, and (6) the literature discussing only the clinical application or implementation of guidelines without presenting original evidence. After the initial screening, discrepancies between the two researchers were resolved through consultation with a third researcher for final adjudication. The quality of the guidelines was evaluated through the Appraisal of Guidelines for Research and Evaluation (AGREE II) tool, which systematically evaluates the guideline quality. Supplementary Material presents detailed evaluation standards and procedures. The AGREE II tool comprises 23 items across six domains: scope and purpose, stakeholder involvement, rigor of development, clarity and presentation, applicability, and editorial independence. Additionally, expert consensus was evaluated based on standards set by the JBI Evidence‐Based Healthcare Center in Australia, which includes seven evaluation items. The level of evidence for all included studies was classified according to the JBI Evidence Pregrading System (2014 edition), and the extracted evidence was reviewed and discussed by the research team.

##### 2.2.1.4. Integration and Content Analysis of Recommendations

Researchers (YW and JY) extracted the original recommendations related to PA for overweight and obese EC patients from guidelines and expert consensus documents. Subsequently, two other researchers (JG and YC) verified and translated these recommendations. After translation, the opinions were consolidated, and the final version of the recommendations in Chinese was formed. The evidence levels were discussed and classified based on the type of study, ranging from Level 1 (*highest*) to Level 5 (*lowest*). A descriptive analysis of the general data from the guidelines was performed using SPSS software. The consistency of the evaluation results from the three raters was tested based on the Intraclass Correlation Coefficient (ICC). Ultimately, 15 studies were included, comprising 11 guidelines and four expert consensus documents.

### 2.3. Methods—Step 3: Modeling the Practice Program and Outcomes

The core of a complex intervention refers to the detailed, rigorous, and rational design of the implementation plan. Therefore, a method of content validation and revision was employed during the modeling phase. This approach combines expert consultation with in‐depth discussions among the development team on all components of the intervention. The content revision phase primarily aims at making appropriate adjustments to necessary items and ultimately optimizing the intervention plan.

#### 2.3.1. Expert Panel

##### 2.3.1.1. Study Population, Sampling, and Recruitment

This study employed the Delphi expert consultation method to refine the draft intervention plan for PA guidance in overweight and obese EC patients. The experts were conveniently selected from tertiary hospitals or universities in Beijing and other regions, specializing in gynecologic oncology, sports medicine, endocrinology, clinical nursing, nursing management, and related clinical or research fields. Twenty experts were recruited. According to the Delphi expert selection criteria, the inclusion criteria were as follows: (1) a bachelor’s degree or higher, with a mid‐level or higher professional title; (2) at least 10 years of work experience; (3) a background in obesity management, gynecology, oncology, or PA education; and (4) a high level of enthusiasm. Sixteen experts from Beijing, Shaanxi Province, and Henan Province, who met these criteria, were ultimately included.

#### 2.3.2. Results of Modeling Procedure

An expert consultation questionnaire was designed and sent to eligible experts. It included three sections: (1) survey introduction, providing background, objectives, and instructions; (2) main PA guidance plan for overweight and obese EC patients, with items evaluated on a five‐point Likert scale and a section for suggestions; and (3) expert background information, collecting details about their familiarity and criteria. After the first round, items were analyzed, discussed, and revised based on scores and feedback. The revised questionnaire was sent for a second round, and the process ended when consensus was reached on the PA guidance plan.

### 2.4. Ethical Considerations

Additionally, the study received ethical approval from the Institutional Review Board (IRB) of Peking University People’s Hospital (Approval No. 2023PHB183‐001, September 2023).

## 3. Result

### 3.1. Results—Step 1: Preparation

#### 3.1.1. Intervention Development Team

##### 3.1.1.1. Characteristics of the Participants

The intervention group consisted of eight experts (two gynecologic oncologists, two evidence‐based medicine specialists, one kinesiology expert, one endocrinology expert, and one nursing management expert) and one overweight and obese EC patient. The tasks of the intervention group were as follows: (1) to define the theoretical foundation and content framework for the PA plan for overweight and obese EC patients, (2) to identify and review relevant literature for evidence synthesis, and (3) to draft and revise the PA plan for overweight and obese EC patients.

#### 3.1.2. Selection of Theories and Development of a Content Framework

We employed the Pathway for Evidence‐Based Nursing Practice to devise and implement the intervention plan. This process entailed identifying nursing issues, searching for the best evidence, evaluating and integrating this evidence with expert knowledge, and developing and implementing a PA guidance intervention for overweight and obese EC patients. Additionally, we engaged in continuous quality improvement. This methodology provided a robust theoretical foundation for the intervention.

Subsequently, we conducted a descriptive qualitative study to investigate the factors that impede weight management in EC patients. Seventeen overweight or obese EC patients, who were active participants in PA, were interviewed. We collected 75,000 words of transcript data from these interviews. The ages of the participants ranged from 28 to 40 years, with a mean age of 34.88 ± 3.79. Most participants possessed a bachelor’s degree (*n* = 8), followed by those with associate degrees (*n* = 4) and graduate degrees (*n* = 3). One participant had completed middle school education, and another had vocational school education. Concerning employment status, one participant was unemployed, five were office clerks, three worked in the education sector, and two were employed in the media industry (supplementary material).

**FIGURE 2:**
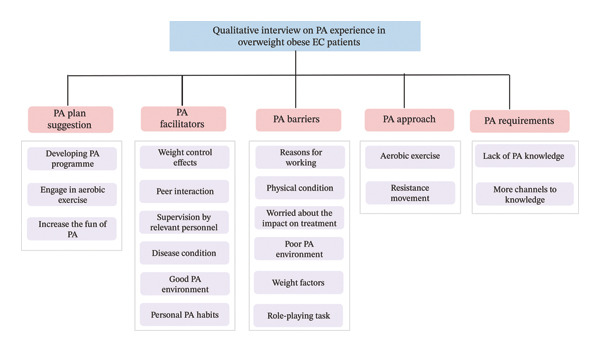
Qualitative interview on physical activity experience in overweight obese endometrial cancer patients. Note: PA: physical activity.

The total duration of the interviews was approximately 485 min, with an average interview duration of 28.53 min per participant. The analysis of the interview data from these 17 overweight and obese EC patients yielded five main themes and 19 subthemes. The main themes included recommendations for PA plans, facilitators, barriers, types of PA, and uncertainties regarding PA (Figure 2).

### 3.2. Results—Step 2: Development of Interventions and Implementation Strategies

#### 3.2.1. Summary of Best Evidence

##### 3.2.1.1. Literature Selection and Characteristics

**FIGURE 3:**
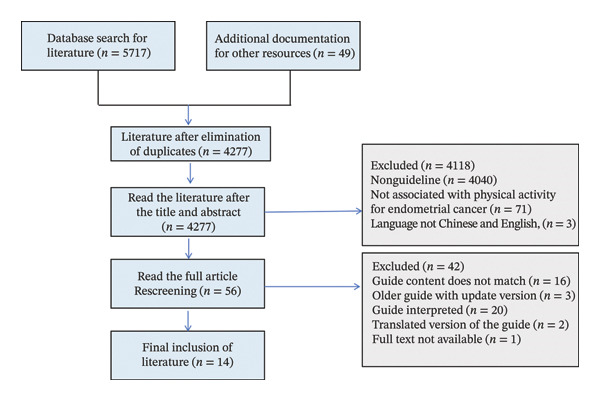
The flow of included literature.

During the literature search, we retrieved a total of 5766 articles. After removing duplicates with EndNote 20, 4277 articles remained. An initial screening based on titles and abstracts yielded 56 articles. Following a full‐text review, 41 articles were excluded, leaving 14 studies for inclusion. These comprised 11 guidelines and three expert consensus papers. The flow of the included literature is depicted in Figure 3, with detailed information available in the reference list and supplementary material.

##### 3.2.1.2. Quality Assessment of Guidelines and Expert Consensus

Eleven guidelines were included in the analysis. Five were rated as Grade B. The other six were rated as Grade A, indicating their high quality and strong reference value. Additionally, three expert consensus papers were evaluated, all of which demonstrated high quality. The results of the quality assessment for both the guidelines and expert consensus are presented in the supplementary material. The ICC ranged from 0.910 to 0.988, indicating satisfactory consistency across all guidelines.

##### 3.2.1.3. Evidence Summary

This study summarized 32 high‐quality pieces of evidence related to PA in overweight and obese women with EC. The evidence was categorized into seven areas: benefits and safety of PA (Evidence 1–4), PA readiness assessment (Evidence 5–8), total PA volume (Evidence 9–14), types of PA (Evidence 15–20), precautions for PA (Evidence 21–23), weight management (Evidence 24–28), and maintenance of PA and health education (Evidence 29–32). A detailed breakdown of these categories is provided in the supplementary material.

### 3.3. Result—Step 3: Modeling the Practice Program and Outcomes

#### 3.3.1. Expert Panel

Based on the established content framework and the synthesized best evidence, we developed a draft of the PA guidance plan. At this stage, we invited relevant experts to refine the plan, leveraging their extensive clinical experience and robust theoretical knowledge.

We carried out two rounds of expert consultations. Twenty experts were given invitations. To be specific, 16 experts agreed to participate. The group covered eight gynecological oncologists, three gynecological nurses, two nursing management experts, one sports training expert, one exercise rehabilitation expert, as well as one endocrinology expert. Following their acceptance, a questionnaire was sent to the 16 experts for collecting information regarding their personal characteristics and revision suggestions. The response rate was 100% for both rounds.

#### 3.3.2. Results of Modeling Procedure

The PA guidance plan for overweight and obese EC patients received approval from experts, who recommended several revisions: (1) renaming “PA readiness assessment” to “risk assessment for PA” or “pre‐activity assessment,” (2) adding more detailed assessment methods, and (3) clarifying methods for determining PA intensity. In response to these suggestions, our team convened a meeting to refine the draft and incorporated the experts’ recommendations into the final version, which is provided in the supplementary material. The detailed content of the expert consultations regarding these modifications is presented in Table 1.

**TABLE 1:** Expert consultation on spendometrial cancerific modifications.

Number	Experts suggest revising the content	Experts suggest modifying the method	Modify the results
1	Level 1 item: The expression content of the basis of the theory of physical activity is unclear	It is rendometrial cancerommended to revise it to physical activity early education	Modify according to expert suggestions
2	Sendometrial cancerondary item: Assessment of physical activity readiness was inaccurate	Risk assessment of physical activity or assessment before physical activity	Modify according to expert suggestions
3	Level 2 item: Inaccurate representation of total physical activity	Increase the frequency, intensity, time, and method of physical activity	Supplement according to expert advice
4	Level item: 2.1.1 The evaluation method is missing, it is rendometrial cancerommended to refine	It is rendometrial cancerommended to add spendometrial cancerific assessment tools	Supplement according to expert advice
5	Level 3 item: 2.1.4 The subjendometrial cancert is not clear	Cancer survivors and cancer patients should be unified	Supplement according to expert advice
6	Level 3 item: 2.2.3 moderate intensity exercise (heart rate 64%–76% maximum heart rate during exercise) was underdescribed	It is rendometrial cancerommended to add the maximum heart rate calculation method of 170‐0.7 ∗ age	Supplement according to expert advice
7	Level 3 item: The determination of physical activity exercise intensity is not clear	How to determine the amount of activity met is the key point in the implementation, it is rendometrial cancerommended to increase the method to determine the intensity of physical activity	Supplement according to expert advice
8	The rendometrial cancerommended population for some tertiary items is unknown	Confirm whether patients are gynendometrial cancerological cancer or endometrial cancer	Modify according to expert suggestions
9	Level 3 item: Active physical activity of at least 6000 steps per day is not rendometrial cancerommended	Step number method to establish daily physical activity is not rendometrial cancerommended, added to daily activities	Deleted according to the expert suggestions
10	The training is not accurate	Modified to the resistance training	Modify according to expert suggestions
11	Inaccurate description of physical activity	Physical activity and physical activity have distinction, need unified	Modify according to expert suggestions
12	Level 3 item: 2.4.3 The presence of caregivers or professionals is not spendometrial cancerific and difficult to implement	It is revised to set up a physical activity supervision and guidance management team to assist patients in their exercise	Modify according to expert suggestions
13	Level III item: 2.5.2 Weight units should be commonly used in China	It is rendometrial cancerommended to change the pound to kg	Modify according to expert suggestions

Following this, in line with the next stage of the MRC framework, a pilot single‐center, prospective cohort study was conducted. The objective of this phase was to evaluate the effectiveness of the PA guidance plan and to further refine the intervention strategy.

**FIGURE 4:**
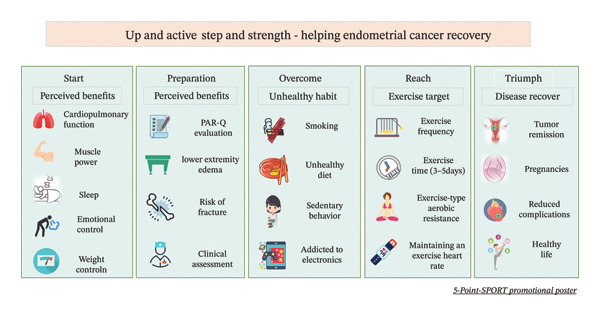
5‐Point campaign poster for overweight obese endometrial cancer patients.

**FIGURE 5:**
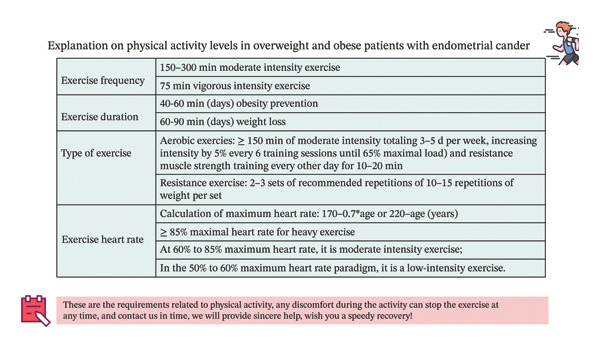
Explanation on physical activity levels in overweight and obese patients with endometrial cancer.

We produced a guidance manual, which includes the benefits of PA, for healthcare professionals, preparations before exercise, recommended types of activities, and precautions during PA. Additionally, we developed a 5‐point‐SPORT promotional poster for patients featuring the steps: Start‐Perceived benefits, Progress‐Perceived benefits, Overcome‐Unhealthy habits, Reach‐Exercise target, and Triumph‐Disease recovery (Figure 4). The exercise goals to be achieved were also provided (Figure 5).

## 4. Discussion

To our knowledge, this is the first comprehensive PA intervention protocol specifically designed for overweight and obese EC patients, developed based on the MRC framework for complex interventions. It is also noteworthy that we have developed a dedicated training manual for healthcare providers and a health education handbook for patients [20]. These resources collectively comprise a complete “protocol‐tool‐training” intervention system, providing a robust foundation for future clinical implementation.

### 4.1. Best Evidence Synthesis Analysis

In the process of synthesizing evidence for the development of a PA intervention protocol for overweight or obese EC patients, this study meticulously accounted for the variations between the physical characteristics of the Chinese population and the international BMI classification criteria. A dual‐track BMI standard was employed for literature screening to enhance the evidence’s comprehensiveness and applicability. Specifically, the Chinese literature was evaluated using the Chinese criteria (overweight: BMI 24.0–27.9 kg/m^2^; obesity: BMI ≥ 28.0 kg/m^2^) [26, 27], whereas the English literature was assessed according to internationally recognized standards (overweight: BMI 25.0–29.9 kg/m^2^; obesity: BMI ≥ 30.0 kg/m^2^) [28]. This method effectively mitigated the risk of evidence omission due to inconsistent standards and improved the systematic nature and representativeness of the evidence base. The adoption of this dual‐standard strategy not only demonstrates respect for the physiological differences among populations but also enhances the cross‐cultural applicability of the research findings, thus laying a scientific groundwork for the subsequent creation of a clinically operable and culturally adaptable intervention protocol.

### 4.2. Scientific Rationale of the Exercise Intervention for Overweight/Obese EC Patients

In contrast to traditional exercise prescriptions, which are often empirically developed, the current intervention was systematically designed following the MRC framework. This approach ensured both scientific rigor and practical applicability. An interdisciplinary team, including experts in gynecology, rehabilitation medicine, nutrition, and evidence‐based nursing, was formed during the development phase. Using the “Evidence‐Based Nursing Practice Pathway” as the theoretical foundation, the team initially synthesized the best available evidence. Subsequent phases involved conducting in‐depth interviews with patients to identify specific barriers to PA in this demographic. These barriers included obesity‐related comorbidities and fatigue associated with cancer treatment, as well as individual exercise preferences. The integration of evidence, theoretical frameworks, and patient needs ensured that the intervention was well‐aligned with real‐world clinical contexts. This alignment provided a strong foundation for high acceptability and potential adherence in future implementations. The intervention’s development culminated in achieving clinical consensus through a Delphi expert consultation process. This process not only validated but also strengthened the safety and scientific robustness of the program, resulting in a rigorously developed intervention system.

### 4.3. Feasibility Analysis of the Exercise Intervention for Overweight/Obese EC Patients

While current clinical guidelines underscore the benefits of PA for EC patients [29, 30], they often lack specificity concerning the type, duration, and intensity of exercise, especially for overweight or obese individuals within this demographic. The intervention developed in this study establishes precise criteria for the benefits and safety of PA, detailing exercise type, frequency, duration, and intensity, tailored to patients across various BMI levels and treatment phases. This approach provides standardized guidance for healthcare providers. Additionally, a descriptive study was conducted among key stakeholders, specifically EC patients, within the Chinese cultural context to identify factors influencing PA engagement. Supported by a health education handbook created in conjunction with the intervention, the program employs patient‐friendly explanations of exercise principles and safety precautions to enhance understanding and mitigate concerns about exercise safety. These efforts aim to increase the acceptance of the intervention among EC patients. By integrating these measures, the study bridges the gap between theoretical, evidence‐based recommendations, and practical clinical application, offering a viable strategy for enhancing outcomes and quality of life in overweight/obese EC patients.

### 4.4. Limitations and Future Directions

This study faces significant limitations. Firstly, the intervention was primarily shaped by professional recommendations derived from clinical experience rather than empirical data. It did not involve consultations with a broader spectrum of stakeholders, including caregivers, general practitioners, and social workers, to comprehensively assess the needs and barriers encountered in guiding or supporting EC patients in PA. Consequently, future qualitative research should focus on the perspectives and requirements of various stakeholders involved in advising and caring for EC patients to optimize the intervention’s effectiveness. Secondly, although the intervention was evidence‐based, it has not yet incorporated advanced technological components, such as smart healthcare solutions. Future versions could integrate artificial intelligence (AI) technology to enhance the intervention through more sophisticated digital and intelligent management approaches, thus improving its practicality in clinical settings. Lastly, the proposed intervention requires empirical validation through feasibility studies and randomized controlled trials, particularly to evaluate its long‐term effects on weight management, quality of life, and clinical outcomes. Additionally, practice‐based data should be gathered to produce high‐quality original research findings.

## 5. Conclusions

Guided by the MRC framework, this study developed an evidence‐based PA guidance intervention strategy for overweight and obese EC patients through three phases: preparation, intervention and implementation strategy formulation, and modeling processes and outcomes. The goal is to implement the intervention in clinical practice to enhance weight management in EC patients, thereby improving tumor remission rates and pregnancy outcomes for patients undergoing fertility preservation treatment. Following this, a pilot prospective cohort study was conducted to assess the effectiveness of the PA guidance plan and further refine and optimize the intervention strategy.

## Author Contributions

Yongli Wang: writing–original draft, visualization, validation, endometrial cancert administration, methodology, investigation, formal analysis, data curation, and conceptualization. Jingying Yang: writing–original draft, visualization, endometrial cancert administration, methodology, investigation, formal analysis, data curation, and conceptualization. Jianliu Wang: writing–review and editing, endometrial cancert administration, and methodology. Jingjing Gong: investigation, formal analysis, and data curation. Yuanyuan Gong: investigation, formal analysis, and data curation. Qingran Li: investigation and data curation. Dandan Yang: investigation, formal analysis, and data curation. Hongmei Zhu: writing–review and editing, methodology, and conceptualization. Peng Liu: writing–review and editing, methodology, and investigation. Yiqian Chen: data curation, investigation, and software. Yingchao Xiong: data curation, formal analysis, and methodology. Xiaodan Li: writing–review and editing, supervision, methodology, investigation, funding acquisition, formal analysis, and conceptualization.

## Funding

Funding for this trial was provided by the National Key Tendometrial cancerhnology Research and Developmental Program of China (Program Nos. 2022YFC2704400 and 2022YFC2704405).

## Disclosure

The funder had no involvement in the design and conduct of the study; the collection, management, analysis, and interpretation of the data; the preparation, review, or approval of the manuscript; or the decision to submit the manuscript for publication. All authors approved the final version for submission. The funding source had no role in any of the dendometrial cancerisions taken in planning and conducting the endometrial cancert or publishing the results.

## Conflicts of Interest

The authors declare no conflicts of interest.

## Supporting Information

Table S1: Interview Guide for Physical Activity Experiences of Overweight and Obese Patients with Endometrial Cancer; Table S2: PubMed Search Strategy Guidelines; Table S3: Demographic information of interview subjects of overweight obese endometrial cancer patients (*n* = 17); Table S4: Inclusion of information on guidelines and expert consensus diseases; Table S5: Results of the evaluation of the quality of the guidelines (*n* = 11); Table S6: Qualitative evaluation of expert consensus; Table S7: Summary entry of the best evidence of physical activity in overweight and obese endometrial cancer; Table S8: Physical activity instruction program for overweight and obese endometrial cancer patients.

## Supporting information


**Supporting Information** Additional supporting information can be found online in the Supporting Information section.

## Data Availability

The data that support the findings of this study are available from the corresponding author upon reasonable request.
