# Supplementary material for: Developing a Complex Intervention Plan for Physical Activity in Overweight and Obese Endometrial Cancer Patients: A Multimethod Study
Source: J Nurs Manag. 2026 Mar 4;2026:3951371. doi: 10.1155/jonm/3951371 (PMC12961224; doi:10.1155/jonm/3951371)
Supplement: Supplementary file 1 — Supporting Information Additional supporting information can be found online in the Supporting Information section. [file JONM-2026-3951371-s001.docx]

**Supplementary Material**

**Table S1 Interview Guide for Physical Activity Experiences of Overweight and Obese Patients with Endometrial Cancer**

| Formal interview outlines |
| --- |
| 1)What is your current lifestyle like?  2)What physical activities do you engage in on a daily basis? Please describe these activities in detail, including the types and frequency.  3)If a physical activity guidance plan were to be developed for you, what elements would you like to see included in this plan?  4)What factors do you think might hinder your ability to consistently engage in physical activities?  5)What factors do you think might facilitate the implementation of physical activities for you?  6)Do you have any other questions related to physical activities? |

**Summary of the best evidence for physical activity in overweight obese endometrial cancer patients**

**Search Source**

(1) Guideline websites

The National Guideline Clearinghouse (NGC) in the USA, Guideline International Network (GIN), Scottish Intercollegiate Guidelines Network (SIGN), National Institute for Health and Clinical Excellence (NICE), New Zealand Guidelines Group (NZGG), Registered Nurses Association of Ontario (RNACO), Canadian Association of Registered Nurses (CRN), and the National Nursing Council of Ontario (NNCO). Registered Nurses Association of Ontario (RNAO), Canadian Medical Association (CMA), Registered Nurses Association of Ontario (RNAO), Joanna Briggs Center (JBCC), Australia, and the National Institute for Health and Clinical Excellence (NICE). CMA, Registered Nurses Association of Ontario (RNAO), Joanna Briggs Center (Australia), China Guideline Clearinghouse (CGC), and Medical Pulse.

(2) Websites of professional associations

China Anti-Cancer Association, National Comprehensive Cancer Network (NCCN), American Cancer Society (ACS), Exercise and SportScience Australia (ESSA), and the National Cancer Society (NCS). Australia (ESSA), American College of Sports Medicine (ACSM), World Cancer Research Fund/American Institute for Cancer Research, WCRF/AIC. Research, WCRF/American Institute for Cancer Research (WCRF/AICR), British Gynaecological Cancer Society (BGCS), European Society of Gynaecological Oncology (ESGO).

(3) Domestic and international literature databases:

Cochrane Library, JBI Evidence-Based Health Care Database, Clinicalkey for Nursing, Medline, Pubmed, Embase, CINAHL, China National Knowledge Infrastructure (CNKI), Chinese Biomedical Literature Database (Chinese Biomedical Literature Database). (China National Knowledge Infrastructure, CNKI), Chinese Biological Medical Literature (CBM), Wanfang database, and so on.

Taking the "PubMed" database search as an example:

Table S2 PubMed Search Strategy Guidelines

| #1 | "Exercise Therapy"[MeSH Terms] |
| --- | --- |
| #2 | "Exercise"[MeSH Terms] |
| #3 | "physical activity"[Title/Abstract] |
| #4 | #1 or #2 or #3 |
| #5 | "endometrial neoplasms"[MeSH Terms] |
| #6 | "uterine neoplasms"[MeSH Terms] |
| #7 | "neoplasms"[MeSH Terms] |
| #8 | "cancer survivors"[Title/Abstract] |
| #9 | #5 or #6 or #7 or #8 |
| #10 | "overweight"[MeSH Terms] |
| #11 | "obesity"[MeSH Terms] |
| #12 | "weight control"[Title/Abstract] |
| #13 | #10 or #11 or #12 |
| #14 | #9 or #13 |
| #15 | guideline[Publication Type] |
| #16 | practice guideline[Publication Type] |
| #17 | #15 or #16 |
| #18 | #4 AND #14 AND #17 |

Table S3 Demographic information of interview subjects of overweight obese endometrial cancer patients (n=17)

| Number | Age（years） | Educational level | Type of job |
| --- | --- | --- | --- |
| P1 | 33 | College | Office Clerk |
| P2 | 36 | Middle school | Office Clerk |
| P3 | 34 | College | self employed |
| P4 | 37 | master degree | editor |
| P5 | 34 | master degree | teacher |
| P6 | 39 | College | Education Industry |
| P7 | 29 | College | Office Clerk |
| P8 | 30 | College | Property service staff |
| P9 | 32 | College | Media workers |
| P10 | 40 | College | Unemployed personnel |
| P11 | 40 | High school | Fast selling goods business |
| P12 | 37 | College | Office Clerk |
| P13 | 35 | College | teachers |
| P14 | 33 | master degree | salesperson |
| P15 | 40 | College | Office Clerk |
| P16 | 36 | College | News media workers |
| P17 | 28 | College | Nursing |

**Table S4 Inclusion of information on guidelines and expert consensus diseases**

| Inclusion of literature | Published/year | | Publishing organisations | Literature sources | Literature theme | type |
| --- | --- | --- | --- | --- | --- | --- |
| Rock CL^[1]^et al | | 2022 | ACS | Pubmed | PA for cancer survivors | guideline |
| Denlinger CS^[2]^et al | | 2018 | NCCN | Pubmed | PA for cancer survivors | guideline |
| Segal R^[3]^et al | | 2017 | CCO | Pubmed | PA for cancer survivors | guideline |
| Campbell KL^[4]^et al | | 2019 | ACSM | Pubmed | PA for cancer survivors | guideline |
| Woopen H^[5]^et al | | 2022 | GCIG | Pubmed | Long-term management of gynaecological cancer patients | guideline |
| Piercy KL^[6]^et al | | 2018 | HHS | Pubmed | PA for all | guideline |
| PA Guidelines for the Chinese Population^[7]^ | | 2021 | National Health Commission of the People’s Republic of China | CNKI | PA for all | guideline |
| Yumuk V^[8]^et al | | 2015 | EASO | Pubmed | Overweight and obesity weight management | guideline |
| NICE^[9]^ | | 2023 | NICE | Medical Pulse Website | Overweight and obesity weight management | guideline |
| [Ranjan, P](https://webofscience.clarivate.cn/wos/author/record/1972104)^[10]^et al | | 2022 | AIIMS-DST | Web of science | Management of overweight and obesity in middle-aged women | guideline |
| Chinese Guidelines for Medical Nutrition Therapy of Overweight/Obesity^[11]^ | | 2021 | Nutrition and Metabolic Management Branch, China Association for the Promotion of International Exchange in Health Care (CPIEHC) | Medical Pulse Website | Overweight and obesity weight management | guideline |
| Expert consensus on exercise therapy for patients with malignant tumours in China^[12]^ | | 2022 | Oncology Nutrition Committee of the Chinese Anti-Cancer Association | CNKI | PA in patients with malignant tumours | expert consensus |
| Expert consensus on weight management processes for overweight or obese people^[13]^ | | 2021 | Chinese Medical Association Health Management Branch | Medical Pulse Website | Overweight and obesity weight management | expert consensus |
| Expert consensus on obesity prevention and treatment in China^[14]^ | | 2022 | Chinese Society of Nutrition, Obesity Prevention and Control Branch | CNKI | Obesity prevention and treatment | expert consensus |

Note: PA:Physical Activity;

Relevant Literature

[1] MCCULLOUGH M L, PATEL A V, PATEL R, et al. Body mass and endometrial cancer risk by hormone replacement therapy and cancer subtype [J]. Cancer Epidem Biomar, 2008, 17(1): 73-9.

[2] DASHTI S G, CHAU R, OUAKRIM D A, et al. Female Hormonal Factors and the Risk of Endometrial Cancer in Lynch Syndrome [J]. JAMA, 2015, 314(1): 61-71.

[3] ASANOMA K, YAHATA H, OKUGAWA K, et al. Impact of obesity on robotic-assisted surgery in patients with stage IA endometrial cancer and a low risk of recurrence: An institutional study [J]. J Obstet Gynaecol Re, 2022.

[4] KAAKS R, LUKANOVA A, KURZER M S. Obesity, endogenous hormones, and endometrial cancer risk: a synthetic review [J]. Cancer Epidemiol Biomarkers Prev, 2002, 11(12): 1531-43.

[5] RAGLAN O, KALLIALA I, MARKOZANNES G, et al. Risk factors for endometrial cancer: An umbrella review of the literature [J]. Int J Cancer, 2019, 145(7): 1719-30.

[6] NEAD K T, SHARP S J, THOMPSON D J, et al. Evidence of a Causal Association Between Insulinemia and Endometrial Cancer: A Mendelian Randomization Analysis [J]. Jnci-J Natl Cancer I, 2015, 107(9).

[7] BOUWMAN F, SMITS A, LOPES A, et al. The impact of BMI on surgical complications and outcomes in endometrial cancer surgery--an institutional study and systematic review of the literature [J]. Gynecol Oncol, 2015, 139(2): 369-76.

[8] KOSKAS M, UZAN J, LUTON D, et al. Prognostic factors of oncologic and reproductive outcomes in fertility-sparing management of endometrial atypical hyperplasia and adenocarcinoma: systematic review and meta-analysis [J]. Fertil Steril, 2014, 101(3): 785-94.

[9] SCHMANDT R E, IGLESIAS D A, CO N N, et al. Understanding obesity and endometrial cancer risk: opportunities for prevention [J]. Am J Obstet Gynecol, 2011, 205(6): 518-25.

[10] COURNEYA K S, KARVINEN K H, CAMPBELL K L, et al. Associations among exercise, body weight, and quality of life in a population-based sample of endometrial cancer survivors [J]. Gynecol Oncol, 2005, 97(2): 422-30.

[11] CALLE E E, RODRIGUEZ C, WALKER-THURMOND K, et al. Overweight, obesity, and mortality from cancer in a prospectively studied cohort of U.S. adults [J]. N Engl J Med, 2003, 348(17): 1625-38.

[12] AREM H, IRWIN M L. Obesity and endometrial cancer survival: a systematic review [J]. Int J Obes (Lond), 2013, 37(5): 634-9.

[13] VON GRUENIGEN V E, TIAN C, FRASURE H, et al. Treatment effects, disease recurrence, and survival in obese women with early endometrial carcinoma : a Gynecologic Oncology Group study [J]. Cancer, 2006, 107(12): 2786-91.

[14] YUMUK V, TSIGOS C, FRIED M, et al. European Guidelines for Obesity Management in Adults [J]. Obes Facts, 2015, 8(6): 402-24.

**Table S5 Results of the evaluation of the quality of the guidelines (n=11)**

| Inclusion of literature | Standardised score by area/% | | | | | | | | | ≥60％ areas/  count | ≥30％areas/  count | recommended levels |
| --- | --- | --- | --- | --- | --- | --- | --- | --- | --- | --- | --- | --- |
|  | Scope and objective | | participant | rigour | clarity | | Applicable | | independent |  |  |  |
| Rock CL^[1]^et al | | 81.48 | 66.67 | 26.39 | | 75.93 | | 42.59 | 66.67 | 4 | 5 | B |
| Denlinger CS^[2]^et al | | 81.48 | 74.07 | 81.94 | | 90.74 | | 66.67 | 35.71 | 5 | 6 | A |
| Segal R^[3]^et al | | 87.04 | 70.37 | 61.81 | | 62.5 | | 64.29 | 50 | 5 | 6 | A |
| Campbell KL^[4]^et al | | 87.04 | 81.48 | 75.69 | | 38.89 | | 71.43 | 47.62 | 4 | 6 | B |
| Woopen H^[5]^et al | | 96.11 | 86.11 | 84.03 | | 68.06 | | 38.39 | 92.59 | 5 | 6 | A |
| Piercy KL^[6]^et al | | 90.74 | 83.33 | 69.44 | | 68.06 | | 76.19 | 76.19 | 6 | 6 | A |
| National Health Commission of the People’s Republic of China^[7]^ | | 72.22 | 48.15 | 45.14 | | 65.28 | | 42.86 | 82.14 | 3 | 6 | B |
| Yumuk V^[8]^et al | | 94.44 | 64.81 | 86.11 | | 92.59 | | 54.17 | 36.11 | 4 | 6 | B |
| NICE^[9]^ | | 97.20 | 70.37 | 86.11 | | 74.07 | | 69.44 | 45.78 | 5 | 6 | A |
| [Ranjan, P](https://webofscience.clarivate.cn/wos/author/record/1972104)^[10]^et al | | 94.44 | 48.15 | 81.94 | | 92.59 | | 54.17 | 63.89 | 4 | 6 | B |
| Chinese Guidelines for Medical Nutrition Therapy of Overweight/Obesity^[11]^ | | 96.11 | 86.11 | 84.03 | | 68.06 | | 38.39 | 92.59 | 5 | 6 | A |

**Table S6 Qualitative evaluation of expert consensus**

|  | Oncology Nutrition Committee of the Chinese Anti-Cancer Association^[98]^ | Chinese Medical Association Health Management Branch^[99]^ | Chinese Society of Nutrition, Obesity Prevention and Control Branch^[100]^ |
| --- | --- | --- | --- |
| Are the sources of the ideas clearly labelled? | yes | yes | yes |
| Are the ideas sourced from influential experts in the field? | yes | yes | yes |
| Are the ideas presented centred on the interests of the population of interest to the study | yes | yes | yes |
| Are the stated conclusions based on the results of the analysis? Are the ideas presented in a logical manner? | yes | yes | yes |
| Are references made to other existing literature? | yes | yes | yes |
| Are the ideas presented inconsistent with previous literature? | no | no | no |

**Table S7 Summary entry of the best evidence of physical activity in overweight and obese endometrial cancer**

| Order number | | Recommended opinion | The evidence level | |
| --- | --- | --- | --- | --- |
| **1. Benefits and safety of physical activity** | | | | |
| 1 | | Exercise can improve the quality of life of patients with tumors. Among the areas of QOL assessment, exercise showed the most significant improvements in sleep, depression, fatigue, and physical function, as well as role function. | Level 1 | |
| 2 | | Physical activity after the diagnosis of endometrial cancer may improve the survival rate of gynecologic cancers. | Level 1 | |
| 3 | | In some cases, exercise principles and guidelines for similar outcomes (such as exercise and obesity guidelines for obese women treated for endometrial cancer). | Level 5 | |
| 4 | | People with cancer can exercise moderate exercise during or after completion without adversely affecting their treatment. | Level 5 | |
| **2. Physical activity readiness assessment** | | | | |
| 5 | | All cancer patients should be routinely evaluated before performing any physical activity to determine the impact of existing diseases, treatments, etc., on their physical activity and fitness. | Level 1 | |
| 6 | | Additional medical evaluation may be required for morbidly obese gynecological cancer patients to determine the safety of activities in addition to cancer-specific risk. It is recommended to evaluate lower limb lymphedema exercise before vigorous aerobic exercise or resistance training. | Level 1 | |
| 7 | | To assess the risk of fracture in patients receiving hormonal therapy, confirmed osteoporosis, or bone metastasis. | Level 5 | |
| 8 | | Key clinical assessment: weight / body mass index (BMI), blood pressure, functional status, baseline and current activity levels, survivor assessment of physical activity impairment, environment (eg., home, gym, outdoor space, physical safety), financial, physical limitations, time / competitive requirements, motivation level, social support, stress, system assessment, disease status. | Level 1 | |
| **3. Total physical activity** | | | | |
| 9 | | A minimum of 150 to 300 minutes of moderate intensity activity or 75 minutes of intense intensity activity, or an equal combination should be distributed over the course of the week. | Level 1 | |
| 10 | | It is recommended that 45-60 minutes of moderate-intensity activity per day is required to prevent obesity, and that 60-90 minutes of exercise to avoid weight recovery should occur in order to reduce obesity and weight loss. | Level 1 | |
| 11 | | Increasing exercise needs to be gradual to achieve the frequency of exercise for 3 to 5 days a week. A total of 150 min of moderate intensity aerobic exercise (energy metabolism equivalent) is 3 to 6 MET (1 MET = 3.5 ml · kg ¹ · min ¹), increasing the intensity by 5% every 6 sessions until 65% maximum load. recommended resistance muscle strength training one day apart for 10 to 20 minutes. | Level 1 | |
| 12 | | Resistance training should be recommended 2-3 times, 10-15 times each; individualized recommended resistance and strength training is important. Survivors may consider adding weight when 10-15 repeats 3 times becomes easy. | Level 1 | |
| 13 | | The amount of physical activity is proportional to weight loss; overweight and obese individuals should perform moderate intensity exercise at least 150 min per week for weight loss; to achieve 5% weight loss, the weekly exercise time should reach 300 min and the exercise intensity should be moderate-high exercise energy consumption of 2000 kcal / week or more. | Level 1 | |
| 14 | | Active physical activity reached at least 6,000 steps per day. | Level 5 | |
| **4. Mode of physical activity** | | | | |
| 15 | Women with endometrial cancer need increased resistance training to ensure that lean tissue is preserved during weight loss. | | | Level1 |
| 16 | At least 150 minutes of moderate aerobic training a week (e. g., brisk walking) should be combined with exercise three times a week to increase resistance training in muscle strength. | | | Level1 |
| 17 | Core and strength training are important to maintain balance and to reduce fall risk. | | | Level2 |
| 18 | Compared with moderate intensity continuous exercise, HIIT can serve as an effective exercise mode to reduce weight, reduce fat and improve cardiopulmonary function, and it has the advantage of aging. | | | Level2 |
| 19 | Physical activity can be incorporated into activities of daily life, such as brisk walking, gardening or cycling; other activities, such as swimming, where the goal is to take a certain number of steps per day, or climb stairs. | | | Level5 |
| 20 | All exercises should be preceded by a mild aerobic warm-up and stretching exercise. During the days of other exercises, the main muscle groups should be stretched for at least 2 days per week. | | | Level2 |
| **5. Physical activity considerations** | | | | |
| 21 | For those who are sedentary before diagnosis, low-intensity activities such as stretching exercises and short, slow walking should be improved. | | | Level5 |
| 22 | Physical activity and exercise recommendations should be adjusted according to the survivors individual abilities and preferences. | | | Level5 |
| 23 | It is helpful to have nursing staff or professional exercise practitioners present during the exercise process. | | | Level3 |
| **Vi. Weight management** | | | | |
| 24 | All cancer survivors should be encouraged to achieve and maintain a normal BMI in an effort to achieve metabolic health. | | | Level5 |
| 25 | For cancer survivors who are overweight or obese and choose to lose weight, there appear to be no contraindications for moderate weight loss during treatment (i. e., up to 2 pounds per week), and as long as the treating oncologist agrees, weight loss is closely monitored and does not interfere with treatment. | | | Level1 |
| 26 | For individuals with poor exercise compliance, they can use fragmentary time to accumulate many short exercise, and under the same amount of exercise, the weight loss effect is even better than a continuous long exercise. | | | Level1 |
| 27 | The initial goal of weight loss treatment should be to reduce body weight by approximately 10% from baseline. If successful, further weight loss can be attempted with further evaluation. | | | Level2 |
| 28 | When conducting resistance training, select the short-time vigorous exercise of medium to high intensity for the large muscle group within the safe range, with a rest interval of &lt;1 min, which is helpful to increase the skeletal muscle content and strengthen the weight loss effect. | | | Level2 |
| **7. Physical activity maintenance and health education** | | | | |
| 29 | Considering the individual&#039;s current physical health status and their own ability and activity status. Encourage people to reduce inactive time, such as watching TV, using a computer or playing video games. | | | Level5 |
| 30 | Long-term follow-up must include counselling on modifiable lifestyle factors (obesity, alcohol consumption, physical inactivity, healthy diet balance, smoking). | | | Level5 |
| 31 | Adults were encouraged to reach the recommended levels of activity while maintaining body weight. | | | Level5 |
| 32 | It is recommended that, where possible, cancer patients exercise at moderate intensity on a sustained basis as part of their lifestyle in order to maintain quality of life and improvements in muscular and aerobic fitness in the long term. | | | Level5 |

**Table S8 Physical activity instruction program for overweight and obese endometrial cancer patients**

| Physical activity instruction protocol for overweight and obese endometrial cancer patients |
| --- |
| 1. Early education of physical activities  1.1 Benefits of physical activity  1.1.1 Physical activity after the diagnosis of endometrial cancer is beneficial in improving the quality of life and improving physical function.  1.1.2 Physical activity can improve the survival rate of cancer patients.  1.2 Safety of physical activity  1.2.1 Physical activity guidance for patients with endometrial cancer can be used from overweight and obese patients.  1.2.2 Physical activity is not affected by endometrial cancer and its treatment.  2. Physical activity implementation  2.1 Pre-physical activity assessment  2.1.1 Pre-exercise assessments was performed using the Physical Activity Adaptation Questionnaire (PAR-Q) before starting the exercise intervention to assess the impact of disease, treatment, and comorbidities.  2.1.2 Additional medical evaluation may be required for gynecological cancer patients to determine the safety of activities other than cancer-specific wind faces. It is recommended to evaluate the lower limb lymphedema before vigorous physical activity.  2.1.3 Patients receiving hormone therapy, confirmed osteoporosis, or bone metastasis need to assess the risk of fracture.  2.1.4 Key clinical assessment: weight / body mass index (BMI), blood pressure, functional status, baseline and current activity level, physical activity impairment, environment (eg., family, gym, outdoor space, physical safety), financial, physical constraints, time / competitive requirements, motivation level, social support, stress, system assessment, disease status.  2.2 Physical activity appearance rate, time and intensity  2.2.1 The total activity per week should be at least 150 to 300 minutes of moderate intensity activity or 75 minutes of intense intensity activity, or a combination of equal amounts distributed over the course of the week.  2.2.2 It is recommended that 45-60 minutes of moderate-intensity activity per day is required to prevent obesity, and that 60-90 minutes to avoid weight regain in order to reduce obesity and weight loss.  2.2.3 Increasing exercise needs to be gradual to achieve 3 to 5 d a week of 150 min of aerobic moderate intensity, 5% intensity every 6 sessions until 65% maximum load), and one resistance muscle strength training on an alternate day for 10-20 min.  2.2.4 Resistance training in cancer patients should be recommended for repeated 2-3 with 10-15 weights; personalized recommended resistance and strength training is important. When 10-15 three repetitions become easier, patients can consider adding weight.  2.2.5 There is a significant dose-effect relationship for physical activity weight loss. For overweight and obese individuals, at least 150 min moderate physical activity to achieve moderate weight loss; to achieve 5% weight loss, weekly physical activity time should reach 300 min, and physical activity intensity should be medium-high exercise or exercise energy consumption of 2000 kcal weeks or more.  2.2.6 Maximum heart rate at 85% or more, equivalent to high intensity exercise; heart rate control in the maximum rate range of 60% to 85%, equivalent to moderate intensity exercise; heart rate control in the maximum rate range of 50%~60%, which is equivalent to small intensity exercise. Maximum heart rate calculation method: 170-0.7 * age or 220-age (age). If heart rate cannot be detected, physical activity intensity was assessed using the borg scale. The subjective physical feeling in the process of human physical activity can be divided into 6~20 grades, the subjective physical feeling of small intensity physical activity is relaxed (9~10), the subjective physical feeling of moderate intensity physical activity is slightly tired (13~14), and the subjective physical feeling of high intensity physical activity is tired (15~16).  2.3 Physical activity mode  2.3.1 Women with endometrial cancer need to strengthen resistance training to ensure muscle tissue preservation during weight loss.  2.3.2 At least 150 minutes of moderate aerobic training (e. g. brisk walking) should be combined with exercise three times a week to increase muscle strength.  2.3.3 Core and strength training is important for maintaining balance and low fall risk.  2.3.4 For patients with limited time, HIIT can be used as an effective way to reduce weight, fat and improve cardiopulmonary function, and it has the advantage of aging.  2.3.5 Physical activity can be integrated into activities of daily life, such as brisk walking, gardening or cycling; other activities, such as swimming, aim to take a certain number of steps per day, or climb stairs.  2.3.6 All physical activity should be preceded by mild aerobic warm-up and stretching. During the days of other exercises, the main muscle groups should be stretched for at least 2 days per week.  2.4 Notes for physical activity  2.4.3 Set up a physical activity supervision, guidance and management team to assist patients in their exercise. Organize the physical activity-related activities within the group appropriately.  2.4.4 Recent abdominal surgery or local bleeding should immediately stop physical activity and inform the responsible personnel.  2.5 Physical activity and weight control  2.5.1 All patients should be encouraged to achieve and maintain a normal BMI in an effort to achieve metabolic health.  2.5.2 For cancer survivors who are overweight or obese and choose to lose weight, there seems to be no contraindication for moderate weight loss during treatment (i. e. 0.9kg per week), as long as the treating oncologist agrees that weight loss is closely monitored and does not interfere with treatment.  2.5.3 For individuals with poor exercise compliance, they can accumulate several times of short time exercise in spare time. In the case of the same amount of exercise, the weight loss effect is even better than that of a continuous long time exercise.  2.5.4 The initial goal of weight loss treatment should be to reduce body weight by approximately 10% from baseline. If successful, further weight loss can be attempted by further translation estimation.  2.5.5 During resistance training, select short and vigorous exercise of medium to high intensity for large muscle groups within the safe range, with rest interval <1 min, which is help to increase the skeletal muscle content and strengthen the weight loss effect.  3. Physical activity maintenance  3.1 Health education for physical activities  3.1.1 Take into account the individuals current physical health and mobility. Encourage people to reduce inactive time, such as watching TV, using a computer or playing video games.  3.1.2 Long-term follow-up must include counselling on modifiable lifestyle factors (obesity, alcohol consumption, physical inactivity, healthy diet balance, smoking).  3.2 Physical activity follow-up support  3.2.1 Adults are encouraged to reach the recommended level of activity while maintaining body weight. |
| 3.2.2 It is recommended that, where possible, patients with cancer exercise at moderate intensity on a sustained basis as part of their lifestyle in order to maintain quality of life and improvements in muscle and aerobic fitness in the long term. |
